# Phylogeography of *Yersinia ruckeri* reveals effects of past evolutionary events on the current strain distribution and explains variations in the global transmission of enteric redmouth (ERM) disease

**DOI:** 10.3389/fmicb.2015.01198

**Published:** 2015-10-29

**Authors:** Asmine Bastardo, Carmen Ravelo, Jesús L. Romalde

**Affiliations:** ^1^Departamento de Microbiología y Parasitología, CIBUS, Universidad de Santiago de CompostelaSantiago de Compostela, Spain; ^2^Estación de Investigaciones Hidrobiológicas de Guayana, Fundación La Salle de Ciencias NaturalesSan Félix, Venezuela

**Keywords:** phylogeography, *Yersinia ruckeri*, genetic structure, population changes, aquaculture, Bayesian analysis

## Abstract

Phylogeographic patterns and population genetic structure of *Yersinia ruckeri*, the pathological agent of enteric redmouth disease (ERM) in salmonids, were investigated on the basis of concatenated multiloci sequences from isolates of different phenotypes obtained between 1965 and 2009 from diverse areas and hosts. Sequence analyses revealed genetic differentiation among subpopulations with the largest genetic distance occurring between subpopulations of Europe and Canada and/or South America. Bayesian analysis indicated the presence of three ancestral population clusters. Mismatch distribution displayed signatures characteristic of changes in size due to demographic and spatial expansions in the overall *Y. ruckeri* population, and also in the geographically separate subpopulations. Furthermore, a weak signal of isolation by distance was determined. A significant positive correlation between genetic and geographical distances was observed. These results revealed that the population of *Y. ruckeri* has undergone both ancient and recent population changes that were probably induced by biogeography forces in the past and, much more recently, by adaptive processes forced by aquaculture expansion. These findings have important implications for future studies on *Y. ruckeri* population dynamics, on the potential role of genetic structure to explain variations in ERM transmission, and on the effect of past evolutionary events on current estimations of gene flow.

## Introduction

Studies on the evolutionary history of organisms have benefited from the availability of an increasing amount of data, especially multiple whole genome sequences. This fact has led to more accurate reconstructions of phylogenetic relationships within several bacterial species (Pritchard et al., 2000). Dispersal, geographic isolation, drift processes and selection leave their signature in the pattern of molecular diversity of contemporary populations (Avise, 2000). Thus, sequence data provide direct genealogical information that can be efficiently used to estimate phylogenetic relationships and parameters associated with population dynamics.

Despite the limitations of multilocus sequence typing (MLST) and eBURST analysis for the phylogenetic inferences to determine exact relationships between individual isolates (Castro-Nallar et al., 2015), large MLST data set represent a valuable resource from which population level trends can be obtained. Analysis of allele frequencies can facilitate recognition of distinct populations, and the comparisons of allelic diversity among populations are informative since ancient populations are expected to be more diverse than recent populations (Slatkin and Hudson, 1991; van Gremberghe et al., 2011).

Throughout recorded history, *Yersinia ruckeri*, the etiological agent of enteric redmouth disease (ERM), has been spread multiple times from the USA, probably by egg or carrier fish transfers, as the culture of rainbow trout (*Oncorhynchus mykiss*) became more widely practiced in the world (Austin and Austin, 2012). There is a hypothesis that *Y. ruckeri* could have already existed previously in the USA as suggested by the study of isolates recovered from the National Fisheries Center (USA), which showed some of them to be dated before the first reports of isolation by Rucker in the 1950s (Bullock et al., 1978). In addition, an Australian *Y. ruckeri* isolate was also found dating back to the 1960s (Roberts, 1983). On the other hand, *Y. ruckeri* strains in the UK were not reported until 1980s, nevertheless, the first isolations were achieved in the 1970s but the findings were never published (Roberts, 1983).

Although ERM could potentially affect different salmonid fish (mainly rainbow trout), the microorganism has been isolated from non-salmonid and marine fish (Fuhrmann et al., 1983). Mammals, birds, invertebrates, and even humans were considered as possible vectors of the *Y. ruckeri* (Willumsen, 1989; De Keukeleire et al., 2014). This bacterium has been also recovered from feces and sewage sludge and the aquatic environment, including water (Willumsen, 1989). In addition, it has been described as being readily able to form biofilms (Coquet et al., 2002). These biofilms may be a source of recurrent infection in rainbow trout farms.

ERM has been successfully controlled for decades by vaccination. Although formulations of most commercial vaccines are based only on common serotype O1a, different degrees of cross-protection among serotypes have been reported (Stevenson, 1997). However, recently ERM vaccine breakdowns have been described in Europe and the USA mostly attributed to *Y. ruckeri* non-motile and lipase negative strains (biotype 2) (Austin et al., 2003; Fouz et al., 2006; Arias et al., 2007; Calvez et al., 2014). Other epizootics have been reported in Spain, being caused by uncommon serotype O2b in rainbow trout (Romalde et al., 2003), and in Australia and Chile by serotype O1b/biotype 1 strains in vaccinated Atlantic salmon (*Salmo salar*) (Bastardo et al., 2011; Bridle et al., 2012).

*Y. ruckeri* remains as a concern for aquaculture due to the expanding range of both hosts and pathogen across the world. In this study, the genetic structure of a broad geographical range *Y. ruckeri* population was analyzed using MLST data to evaluate the evolutionary past of this pathogen within the context of ERM re-emergence. Such data together with information on pathogen-host associations are critical to understand the dynamics of ERM agent and to form hypothesis concerning its past and future spread.

## Materials and methods

### Data set construction

The data set used in this study consisted of multiloci concatenated sequences of 103 *Y. ruckeri* isolates previously typed into 30 sequence type (ST) and described by a MLST scheme (Bastardo et al., 2012). DNA sequences for 6 housekeeping genes (*gln*A, *gyr*B, Y-HSP60, *rec*A, *dna*J, and *thr*A) and the concatenated sequences were downloaded from the *Y. ruckeri* MLST database hosted on publmlst.org (http://pubmlst.org/yruckeri/). For analyses, each ST was considered as one haplotype, and the 103 isolates of *Y. ruckeri* were considered as one population, which were divided into nine different subpopulations on the basis of the geographical site of isolation as designated in the MLST database (Table 1).

**Table 1:** **Summary information of *Y. ruckeri* isolates used in this study**.

**Location id^a^**	**Origin^a^**	**MLST type^a^^,^^b^**	**Biotype^a^**	**Serotype^a^**	**Host or Source^a^^,^^c^**	**Year**
CA	Canada (5)	ST5, ST15, ST21, ST24, ST25	BT1 (5)	O1a, O1b, O2a, O2b, O4	*O. mykiss* (3) *O. zibethica S. malma*	1965–1980
FN	Finland (3) Norway	ST 2 (2), ST 3, ST 17	BT1 (2) BT2 (2)	O1a (3), O1b	*O. mykiss* (2) *S. salar* (2)	1985–2009
DG	Denmark (4) Germany	ST1, ST20, ST22, ST23, ST25	BT1 (5)	O1a (2), O2a, O2b (2)	*O. mykiss*,(3) *S. trutta A. anguilla*	1983–1989
UK	United Kingdom (8)	ST1 (4), ST2, ST13, ST14, ST30	BT1 (3) BT2 (5)	O1a (6), O1b, O2b	*O. mykiss* (8)	1995–2007
PO	Portugal (21)	ST2 (14), ST3 (3), ST8, ST19, ST26 (2)	BT1 (7) BT2 (14)	O1a (17), O3 (4)	*O. mykiss* (17) sediment (3) water	1994–2006
SF	Spain (4) France	ST2 (4), ST23	BT1 (5)	O1a (4), O2b	*O. mykiss*(5)	1980–2002
PE	Peru (27)	ST1 (7), ST2 (14), ST9 (2), ST10, ST11, ST12, ST28	BT1 (22) BT2 (5)	O1a	*O. mykiss* (27)	2008
CH	Chile (11)	ST1 (2), ST7 (8), ST8	BT1 (11)	O1a (2), O1b (8), O2b	*S. salar* (11)	2008
US	United States (17)	ST1, ST2 (10), ST4, ST6, ST14, ST16 (3),	BT1 (7) BT2 (10)	O1a (4), O1b (10), O2b, O3, O4	*O. mykiss* (6) *S. trutta* (10) *O. tshawytscha*	1965–2006

a When more than one, number of isolates is indicated in brackets.

b Established by Bastardo et al. (2012). Each ST corresponds to one haplotype in this study.

c Host species: Oncorhynchus mykiss, Ondatra zibethica, Salvelinus malma, Salmo salar, Salmo trutta, Anguilla anguilla, Oncorhynchus tshawytscha.

### Phylogenetic and phylogeographical analysis

Phylogenetic relationships among the concatenated sequences (2876 bp) of the 30 haplotypes (equivalent to each ST) were estimated by constructing a maximum-likelihood (ML) tree with PHYML 3.0 (Felsenstein, 1989). For the analysis of genetic structure and haplotype sharing, a haplotype network was constructed using the program NETWORK 4.1 (http://www.fluxus-engineering.com). Concatenated sequences of the six housekeeping genes from all *Y. ruckeri* isolates were previously aligned using the software DNA Alignment 1. 3 (http://www.fluxus-engineering.com), and the output file were imported into NETWORK 4.1 to conduct a network analysis using a median joining algorithm (Bandelt et al., 1999). The network of closely related haplotypes was displayed using the geographical origins.

### Genetic differentiation

Haplotype polymorphism (Hd), nucleotide diversity (π), and average number of pairwise differences (*K*) were calculated to assess the genetic variability using the software DnaSP 5 (Librado and Rozas, 2009). The pairwise genetic differentiation (*F*_ST_ index) (Wright, 1965) was determined employing the program Arlequin3.5 (Excoffier and Lischer, 2010). An analysis of molecular variance (AMOVA) was performed to partition genetic variation among regions of restricted gene flow using Arlequin 3.5. This program was also used to determine the number of migrants *N*_M_ (estimated number of migrants between populations per generation) among subpopulations, assuming a constant migration index.

### Genetic population structure analysis

The genetic population structure of *Y. ruckeri* was reconstructed using a Bayesian Monte-Carlo Markov chain (MCMC) sampling method that was implemented using the Structure software (Pritchard et al., 2000). This algorithm identifies genetically distinct populations assigning individual haplotypes to populations on the basis of allele frequencies, and determines the individual membership coefficient in each probabilistic population. For this analysis, an admixture model and the assumption of correlated allele frequencies among subpopulations were assumed (Falush et al., 2003). The probability of assigning individuals into clusters was estimated using 100,000 burn-in repetitions and a final run of one million MCMC steps. The number of clusters (K) was set from 1 to 15, and all runs were replicated 20 times to test the stability of the results. The most probable number of populations (K) was determined by means of the model value (ΔK) based on the second-order rate of change, with respect to K, in the likelihood distribution (Evanno et al., 2005) employing the program Structure harvester (Earl and von Holdt, 2011).

### Analysis of demographic history

Historical demographic structure of the genetic variation at the concatenated loci sequences was investigated using coalescent-based Tajima's *D*, Fu and Li D^*^ and F^*^, and Fu's *F*_S_ statistics to test the hypothesis that all mutation are selectively neutral (Tajima, 1989; Fu and Li, 1993; Fu, 1997). Additional tests of neutrality were also performed by assessing the haplotype structure using Ramos-Onsins' *R*_2_ (Ramos-Onsins and Rozas, 2000) and Strobeck's *S* tests (Strobeck, 1987). Analyses of demographic expansion were conducted using DnaSP 5. This program evaluates the significance of these analyses comparing the observed statistics to a distribution of values generated with 5000 coalescent simulations.

The demographic history of the populations were examined using the frequency of distribution of number of mismatches between pairwise sequences, and by modeling the expected distributions under the demographic scenarios of population constant size using DnaSP 5. The population expansion and spatial expansion assumptions were also modeled employing Arlequin 3.5. The fitted model was tested statistically by calculating the sum of squared deviation (*SSD*) of the observed data relative to the model. Harpending's raggedness (*r*) index (Harpending, 1994) was determined to quantify the smoothness of mismatch distributions. Confidence intervals for mismatch distribution parameters were obtained by performing 1000 bootstrap replicates.

### Spatial analysis

Geographical coordinates for each isolate were located using Google Earth (http://earth.google.com/). The physical distance between different pair of sampling locations was then calculated using the ARC CALC 3 Spherical Trigonometry Calculator macro (http://www.jqjacobs.net/astro/arc_form.html). The presence of phylogeographic structure was tested for individual allele frequencies in six equally-spaced classes of geographic distances by mean the spatial autocorrelation Moran's *I* index (Moran, 1950) using the software Gedis v1.74 (Peña et al., 2009). Furthermore, to assess the relative influence of drift and gene flow, association between genetic distance and geographic distance was determined employing the Isolation By Distance (IBD) web service v 3.1.6 (Jensen et al., 2005). Significance of the associations was tested with a partial Mantel test using 10,000 randomizations (Mantel, 1967).

## Results

### Phylogenetic and phylogeographical relatedness

**Figure 1:**
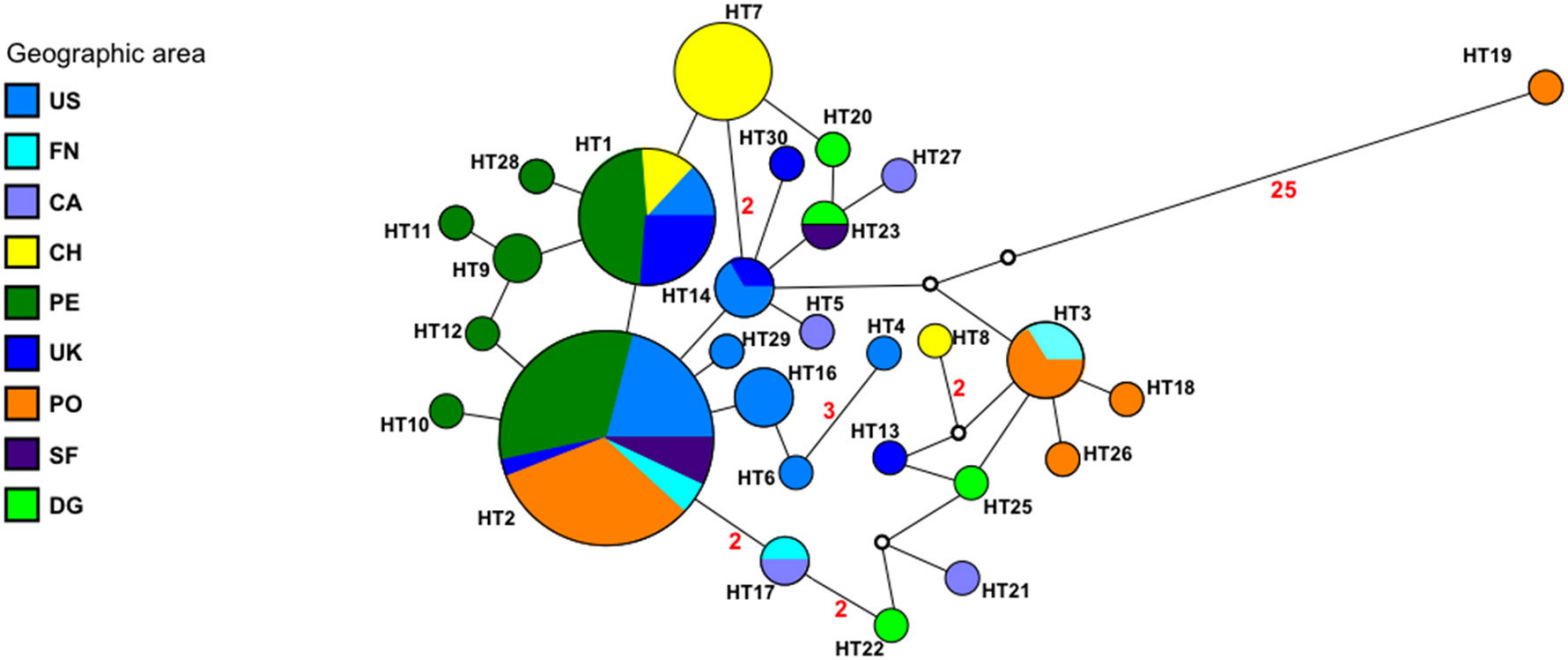
**Median-joining network of *Y. ruckeri* population**. Colors indicate the different geographic area. Circles represent each haplotype (HT). A line between haplotypes represent one mutational step. Numbers on the lines indicate the number of mutational steps greater than one. Open circles represents haplotypes not present in the sample. Radius of the circle is proportional to the number of sequences.

Phylogenetic and phylogeographical analyses included isolates from diverse areas and hosts isolated between 1965 and 2009 (Table 1). The median-joining network constructed using all 103 concatenated sequences, illustrated the mutational relationship of the *Y. ruckeri* haplotypes (Figure 1). All haplotypes (except haplotype 19) differed by less than three mutational steps with a considerable divergence between genomes occurring in different regions. The major groups were separated by one mutational step. Haplotype 2 was the most common interior haplotype found in six different areas, so it is most likely the oldest haplotype. Many haplotypes (13) were tip alleles, being considered as more recently derived and geographically restricted. On the other hand, the majority of haplotypes differed by only one or two mutational steps, suggesting a demographic expansion. Geographical clustering was observed among haplotypes from Portugal (PO), Peru (PE), USA (US), and Chile (CH) subpopulations while the haplotypes present in UK, Denmark/Germany (DG), Finland/Norway (FN), Spain/France (SF), and Canada (CA) were spread into the network. Phylogenetic relationship among all haplotypes using maximum-likelihood was very poorly resolved and not informative probably because the sequence variation contain insufficient phylogenetic signal (data not shown).

### Genetic differentiation

Haplotype diversity ranged from 0.47 to 1.00 among subpopulations with a mean value of 0.79 for the overall population (Table 2). The highest Hd values were observed in CA and DG subpopulations while the lowest value was determined for CH subpopulation. On the other hand, π values ranged to 0.0004 in SF, PE, and CH to 0.0017 in CA and DG, regardless the haplotype diversity observed in each area. Furthermore, genetic differentiation based on haplotype diversity among subpopulations was significant (χ^2^ = 411.87; *P* < 0.001).

**Table 2:** **Statistics of genetic variation observed within the 9 populations of *Y. ruckeri* and results of neutrality tests for different locations studied**.

**Location**	**N**	**HT**	***K***	**Hd**	**π**	***D***	***R*_2_**	***F*_S_**	***S***	D^*^	F^*^
Overall	103	30	2.671 ± 0.89	0.79 ± 0.03	0.0010 ± 0.0002	−2.286^**^	0.056^*^	−20.646^**^	1.00	−6.145^**^	−5.484^**^
CA	5	5	4.800 ± 2.81	1.00 ± 0.12	0.0017 ± 0.0011	0.789	0.131	−1.411	1.00	0.298	0.251
FN	4	3	3.000 ± 1.96	0.83 ± 0.22	0.0011 ± 0.0008	−0.808	0.471	0.731	0.77	−0.808	−0.777
DG	5	5	4.800 ± 2.81	1.00 ± 0.12	0.0017 ± 0.0011	0.789	0.195	−1.411	1.00	0.789	0.830
UK	8	5	1.964 ± 1.23	0.78 ± 0.15	0.0007 ± 0.0005	−0.704	0.191	−1.191	0.93	−0.721	−0.792
PO	21	5	4.380 ± 2.25	0.54 ± 0.11	0.0016 ± 0.0009	−2.037^*^	0.175	3.528	0.08	−3.377^**^	−3.479^**^
SF	5	3	1.200 ± 0.90	0.70 ± 0.21	0.0004 ± 0.0003	−1.048	0.433	−0.186	0.87	−1.048	−1.051
PE	27	7	1.059 ± 0.72	0.65 ± 0.08	0.0004 ± 0.0002	−0.935	0.098	−2.719	0.98	−1.802	−1.799
CH	11	3	1.054 ± 0.75	0.47 ± 0.16	0.0004 ± 0.0003	−1.464	0.232	0.694	0.65	−1.444	−1.634
US	17	7	1.352 ± 0.87	0.71 ± 0.10	0.0005 ± 0.0003	−1.197	0.100	−2.838	0.98	−1.073	−1.274
Cluster I	73		–	–	–	−1.729^*^	0.043	−9.611^**^	1.00	−3.461^**^	−3.450^**^
Cluster II	14		–	–	–	−0.650	0.122	−1.233	0.92	−0.650	−0.965
Cluster III	16		–	–	–	−1.618^*^	0.171	−3.930^**^	0.99	−2.465^*^	−2.592^*^

N, number of sequences analyzed; HT, number of haplotypes; K, average number of pairwise differences; Hd, haplotypes diversity; π, nucleotide diversity; D, Tajima's index; R_2_, Ramos-Onsins' test; F_S_, Fu's statistic; * D* and F*, Fu and Li tests; S, Strobeck's statistic.

* significance at P < 0.05;

** shows significance for Fu's and for Fu and Li tests at P < 0.001, and for Tajima's index at P < 0.01, respectively; Clusters I, II and III, genetic groups defined by genetic population structure analysis.

At the finest scale, pairwise *F*_ST_ values were low among several subpopulation being between −0.0253 and 0.0860, indicative of a high degree of gene flow between different pairs of subpopulations; however these values were not statistically significant (*P* > 0.05) (Table 3). A negative *F*_ST_ value indicates great differences between two random individuals from the same population, rather than between two random individuals from different populations. In contrast, significant pairwise *F*_ST_ values were detected for PE, CH and US compared with the other subpopulations, and for CA respect to UK, indicative of restricted gene flow (*P* < 0.05). The AMOVA results indicated that the highest proportion of the molecular variance (82.67%) could be explained by variations within each subpopulation, while the rest of the variance is explained by the genetic differences among them (Fisher = 0.1733; *P* = 0.000). The net number of migrants between subpopulations per generation *N*_m_ ranged between 0.4 to infinity (Table 3). Moderate *N*_m_ values were found for PE and US respect to the other geographical areas, with the exception of US vs. SF, which were two orders of magnitude larger than the majority (104.6). Infinity values indicated the highest gene flow between pairs of subpopulations. Only the estimated gene flow for CH was low, indicating that this subpopulation is genetically isolated and/or with limited gene flow.

**Table 3:** **Matrix of pairwise genetic differentiation (*F*_ST_) and net number of migrants (*N*_m_) among different sub-populations of *Y. ruckeri***.

	**CA**	**FN**	**DG**	**UK**	**PO**	**SF**	**PE**	**CH**	**US**
CA	–	∞^a^	∞	1.9	12.6	3.1	0.6	0.7	1.1
FN	−0.1422^b^	–	∞	5.3	∞	∞	1.7	0.6	4.5
DG	−0.1215	−0.1262	–	∞	27.5	12.5	1.1	1.2	1.3
UK	0.2126^*^	0.0860	0.0652	–	9.4	72.7	6.3	2.0	2.1
PO	0.0381	−0.0938	0.0179	0.0507	–	∞	3.2	1.4	5.7
SF	0.1379	−0.0253	0.0385	0.0068	−0.0319	–	23.8	0.5	104.6
PE	0.4454^*^	0.2264^*^	0.3199^*^	0.0729	0.1356^*^	0.0201	–	0.5	2.7
CH	0.4212^*^	0.4677^*^	0.2913^*^	0.2001^*^	0.2538^*^	0.4606^*^	0.4891^*^	–	0.4
US	0.3187^*^	0.1006	0.2761^*^	0.1938^*^	0.0801^*^	0.0047	0.1552^*^	0.5354^*^	–

a Upper corner: N_m_ (number of migrants per generation) estimated from F_ST_ values.

b Lower corner: Pairwise F_ST_ values.

* Shows a significant P-value (<0.05).

### Genetic population structure

**Figure 2:**
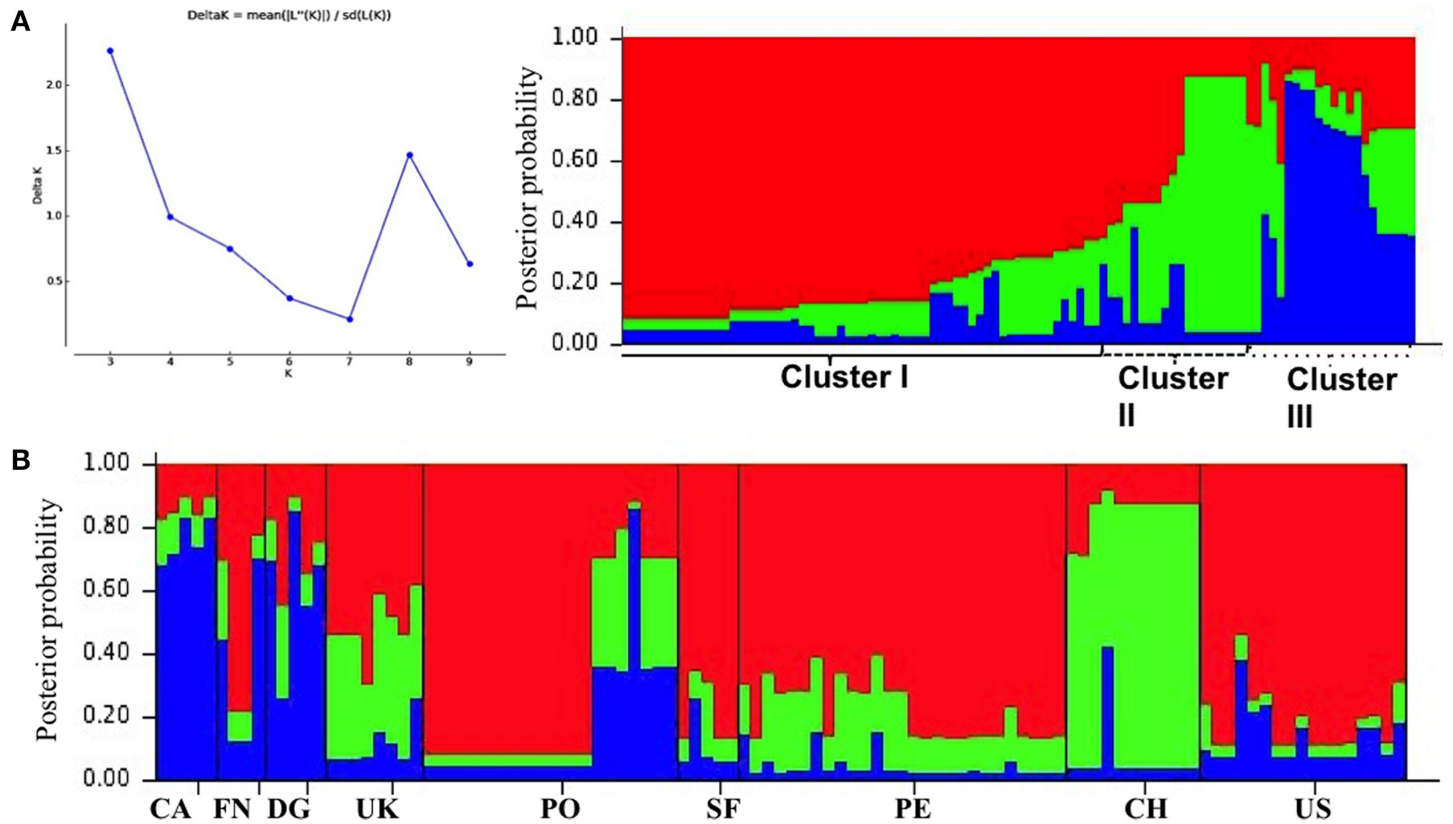
**Structure analysis of 103 *Y. ruckeri* strains at optimal *K* = 3 value**. Each vertical bar represents a single isolate. The height of each color represents the probability of assignment to that cluster. Subpopulations are listed at the bottom and their geographical origins as indicated in Table 1. For each plotting strains ordered by ancestry coefficients **(A)** and by geographic origin **(B)** are showed.

Bayesian analysis of population structure evaluated with Evanno's criterion ΔK, indicated that the data were highly consistent with the presence of three genetic populations or genetic clusters (*K* = 3) (Figure 2A). The majority of isolates from subpopulations FN, PO, SF, PE, UK, DG, and US appear to share similar proportions of ancestry, with posterior probabilities between 0.500 and 0.798 (Figure 2B). These isolates were included in the genetic cluster I (red color) with the exception of six isolates from PE, two isolates from FN, and one isolate from UK. These findings evidence the existence of wide spread processes involved in the current distribution of *Y. ruckeri.* On the other hand, all isolates from CH appeared to be distinct, sharing genetic cluster II (green color) together with other two isolates from PE, and one isolate from UK (posterior probabilities from 0.567 to 0.872) indicating a separate diversification. However, the third genetic cluster (blue color) included in addition to the DG strains, the rest of isolates from FN, US, and CA excluded in clusters I and II (with posterior probabilities values between 0.567 and 0.872). Interestingly, high levels of admixture were present within all groups.

**Figure 3:**
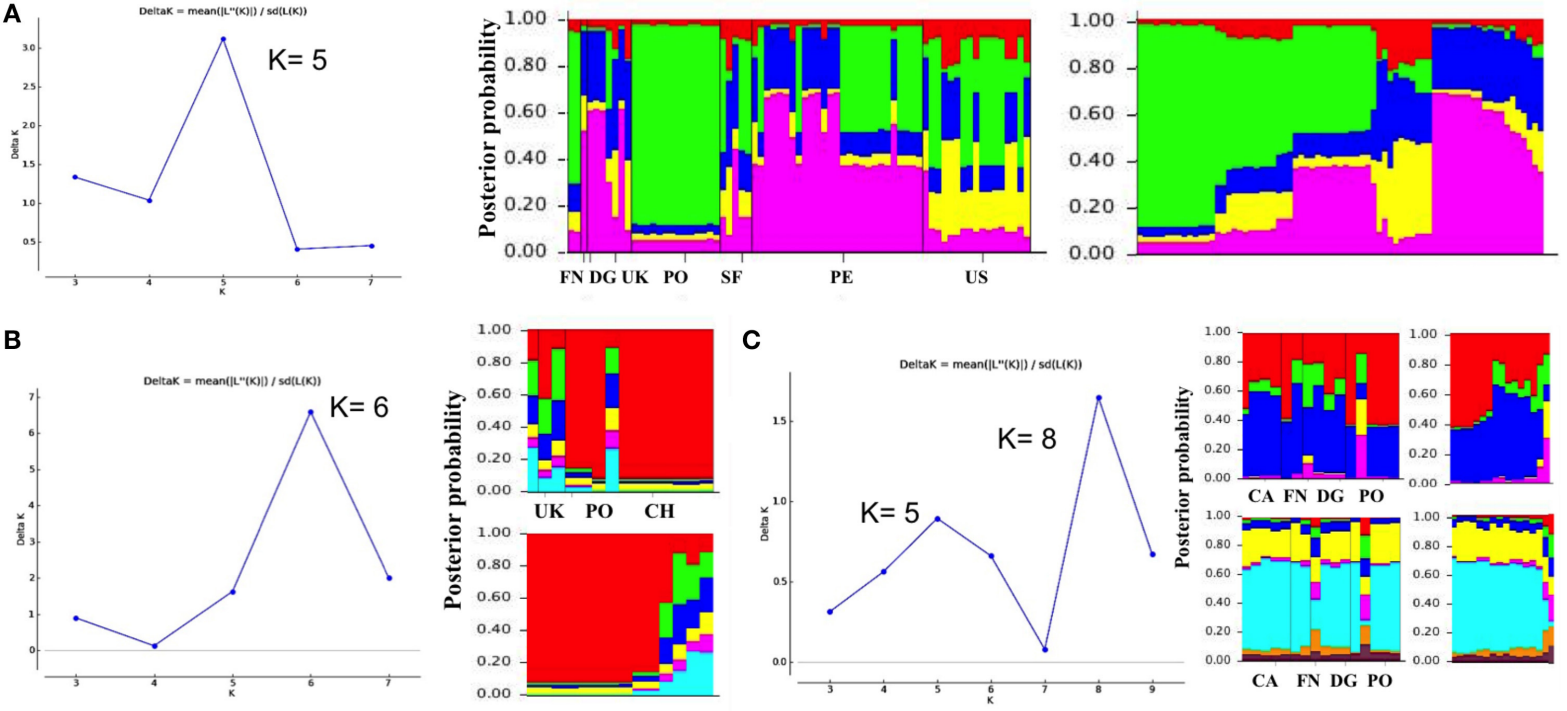
**Populations and subpopulations inferred in clusters I, II, and III by STRUCTURE analyses**. The peak at ΔK represents the most probable number of populations and subpopulations. **(A)** *K* = 5 subpopulations identified within cluster I. **(B)** *K* = 6 subpopulations identified within cluster II, and **(C)** *K* = 8 and *K* = 5 subpopulations determined within the cluster III. Each vertical line in the structure bar plot represents each strain. Different colored segments on the vertical bar represent strains with mixed membership coefficient (maximum 1) to the different population, subpopulation or cluster. For each cluster, plotting ordered by geographic origin, and ancestry coefficients are showed.

To check the substructure within the clusters, the analysis was re-run separately for the strains assigned to each cluster. ΔK determined the presence of genetic subpopulations into each cluster establishing *K* = 5 and *K* = 6 genetic subpopulations in cluster I (Figure 3A) and cluster II (Figure 3B), respectively. Genetic subpopulations in cluster III were supported by two ΔK peaks at *K* = 8 and *K* = 5 but in both scenarios similar proportions of ancestry were retained (Figure 3C). Although relevant geographical differentiations were obtained in concordance with the areas of isolation for the majority of *Y. ruckeri* strains, the isolates found outside of the corresponding genetic subpopulation were indicative of possible migration or introduction processes, gene flow and/or isolation by distance.

An AMOVA conducted by partitioning variation among and within the three clusters revealed that 59.1% of the total variation was attributed to within-population differences (F_CT_ = 0.3504; *P* = 0.000), 31.8% of the variation was associated to the haplotype frequencies among the 3 clusters (F_SC_ = 0.1424; *P* = 0.000), whereas the remaining 9.1% of variation was attributed to within subpopulation-clusters haplotype differences (F_ST_ = 0.4414; *P* = 0.000). Nei's G_ST_ and *N*_m_ (1973) were also determined to examine the haplotype pairwise differentiation and gene flow among the clusters (I, II and III), respectively. High levels of genetic differentiation (*P* < 0.05) was detected among the three clusters with *F*_ST_ between 0.0886 and 0.1309, while the estimation of gene flow showed *N*_m_ values from 1.9 to 2.3 between populations, supporting limited genetic flow by genetic isolation of clusters.

### Demographic history

Tajima's *D* and Fu and Li's F^*^ and D^*^ neutrality tests for the overall population analyzed had significant negative values (Table 2). These results allowed the rejection of the neutral model in the *Y. ruckeri* population as a result of relatively recent population expansion. Fu's *F*_S_ was also negative (*F*_S_ = −20.646; *P* < 0.001), which occurs when an excess of rare haplotypes are present, indicating population expansion or genetic hitchhiking events. Ramos-Onsins' *R*_2_ statistic and Strobeck's *S* index determined that the total population has significant positive *R*_2_ (0.056) and high *S* (1.00) values supporting also possible population expansion. However, FN and PO subpopulations showed positive but non-significant *F*_S_ values, indicating natural selection or population growth. Positive D^*^ values exhibited by CA and DG subpopulations suggest balancing selection, although these values were no significant. When neutrality was tested within each genetic cluster (I, II, and III), the results indicated similar conditions to those observed for the overall population (Table 2).

**Figure 4:**
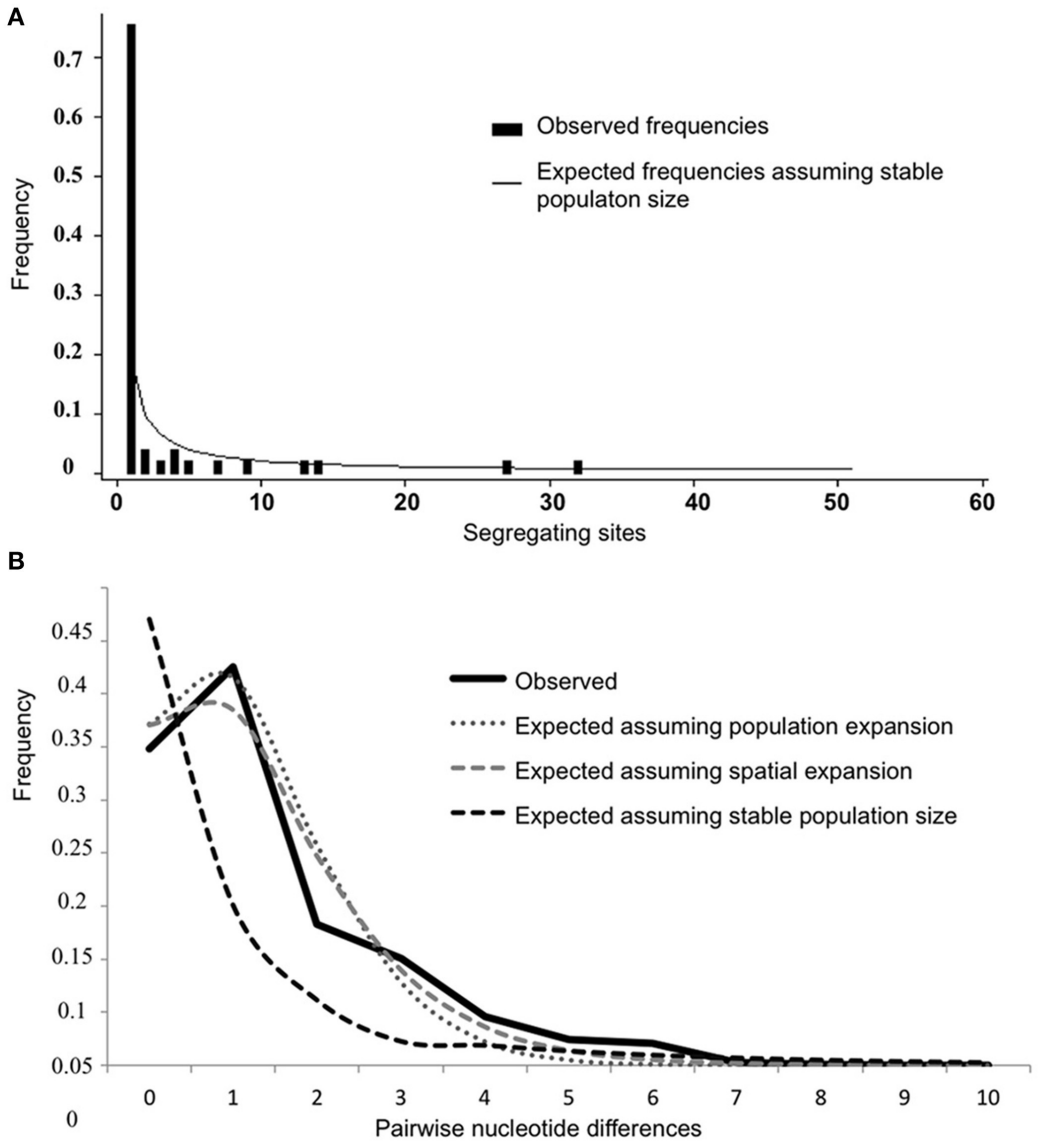
**Population expansion signals in *Y. ruckeri* sequence data. (A)** Site-frequency spectrum indicating excess of singleton mutations in sequences. Spectrum compares observed frequencies of segregating sites to expected distribution under the null hypothesis of no population change. **(B)** Mismatch distribution of observed frequencies of pairwise difference among concatenates sequences and expected frequencies, under neutral model of evolution given the null hypothesis of no population change, population expansion, and spatial expansion.

The mismatch distribution of pairwise nucleotide differences in concatenated sequences of the overall *Y. ruckeri* population showed a smooth unimodal distribution, characteristic of a large population expansion (Figure 4A). The *SSD* statistic and raggedness index values were low, supporting these results (Table 4). Study-wide site-frequency spectra revealed an excess of singleton mutation when compared with expected frequencies under a stable population size (Figure 4B). Similar patterns were observed in the individual distribution for each subpopulation except for FN, UK, and CA, which exhibited a more ragged distribution (data not shown). The FN population differed significantly from the modeled distribution for an expanding population (*P* = 0.04) with high raggedness value (*r* = 0.9722, *P* = 0.05) indicating constant population at equilibrium, although raggedness *P* value supported spatial expansion (*P* = 0.12). Furthermore, the mismatch distribution for each cluster (I, II, and III) also showed a unimodal distribution typical of demographic expansion, supported by the low values for the *SSD* statistic and raggedness index (Table 4).

**Table 4:** **Results of Mismatch distribution analyses for different locations *Y ruckeri* studied**.

**Location**	**N**	***SSD*^†^**	***SSD*^‡^**	***r***	**τ^†^**	**τ^‡^**
Overall	103	0.0802	0.0068	0.0068	1.10 (0.65–1.58)	1.13 (0.67–0.12)
CA	5	0.0870	0.0870	0.2800	4.93 (1.56–7.73)	4.93 (1.27–7.45)
FN	4	0.3278^*^	0.3137^*^	0.9722^*^^†^	3.45 (0.89–6.11)	3.47 (0.67–69)
DG	5	0.0393	0.0393	0.1200	2.74 (0.77–7.98)	2.73 (0.91–8.63)
UK	8	0.0307	0.0280	0.1300	2.51 (0.31–5.36)	2.22 (0.69–5.35)
PO	21	0.0869	0.0236	0.2291	5.19 (0.42–91.19)	3.67 (0.00–9.28)
SF	5	0.0062	0.0052	0.0050	1.57 (0.00–2.91)	1.52 (0.00–3.37)
PE	27	0.0011	0.0010	0.0471	1.08 (0.42–1.87)	0.82 (0.28–2.23)
CH	11	0.0319	0.0295	0.1743	0.15 (0.00–1.58)	4.69 (0.00–124.0)
US	17	0.0132	0.0138	0.1074	0.15 (0.00–1.59)	4.69 (0.00–124.0)
Cluster 1	73	0.0019	0.0013	0.0557	1.00 (0.00–53.5)	0.54 (0.35–2.40)
Cluster II	14	0.0464	0.0355	0.1331	3.89 (0.00–6.416)	2.61 (0.86–6.43)
Cluster III	16	0.0091	0.0092	0.0238	4.44 (2.40–6.69)	4.12 (1.89–5.73)

N, number of sequences analyzed; r, SSD, mismatch distribution; Raggedness index; τ, divergence time;

† , under demographic expansion model;

‡ , under spatial expansion model;

* significance at P < 0.05.

The mismatch distribution parameter, τ (the time in mutational steps per generation since the modeled expansion event), from the raggedness calculation, was 1.10 (95% CI: 0.65, 1.58) for the demographic expansion, and 1.13 (95% CI: 0.67, 0.12) for the spatial expansion for the entire population of *Y. ruckeri* (Table 4). The divergence times were variable for subpopulations founding highest estimations in PO and CA for the demographic expansion, and in CH and US for the spatial expansion. On the other hand, for cluster I τ was estimated at 1.00 (95% CI: 0.00, 53) and 0.54 (95% CI: 0.35, 2.40) mutational steps for both population expansion and spatial expansion, respectively. However, τ parameters were higher for cluster II and cluster III, showing similar τ values for demographic and spatial expansion (τ values between 2.61; 95% CI: 0.86, 6.43 and 4.44; 95% CI: 2.40, 6.69). These findings together provide evidence for demographic and spatial expansion in *Y. ruckeri* population occurring at different times, regardless that the rate of evolution could be similar among subpopulations.

### Spatial analysis

**Figure 5:**
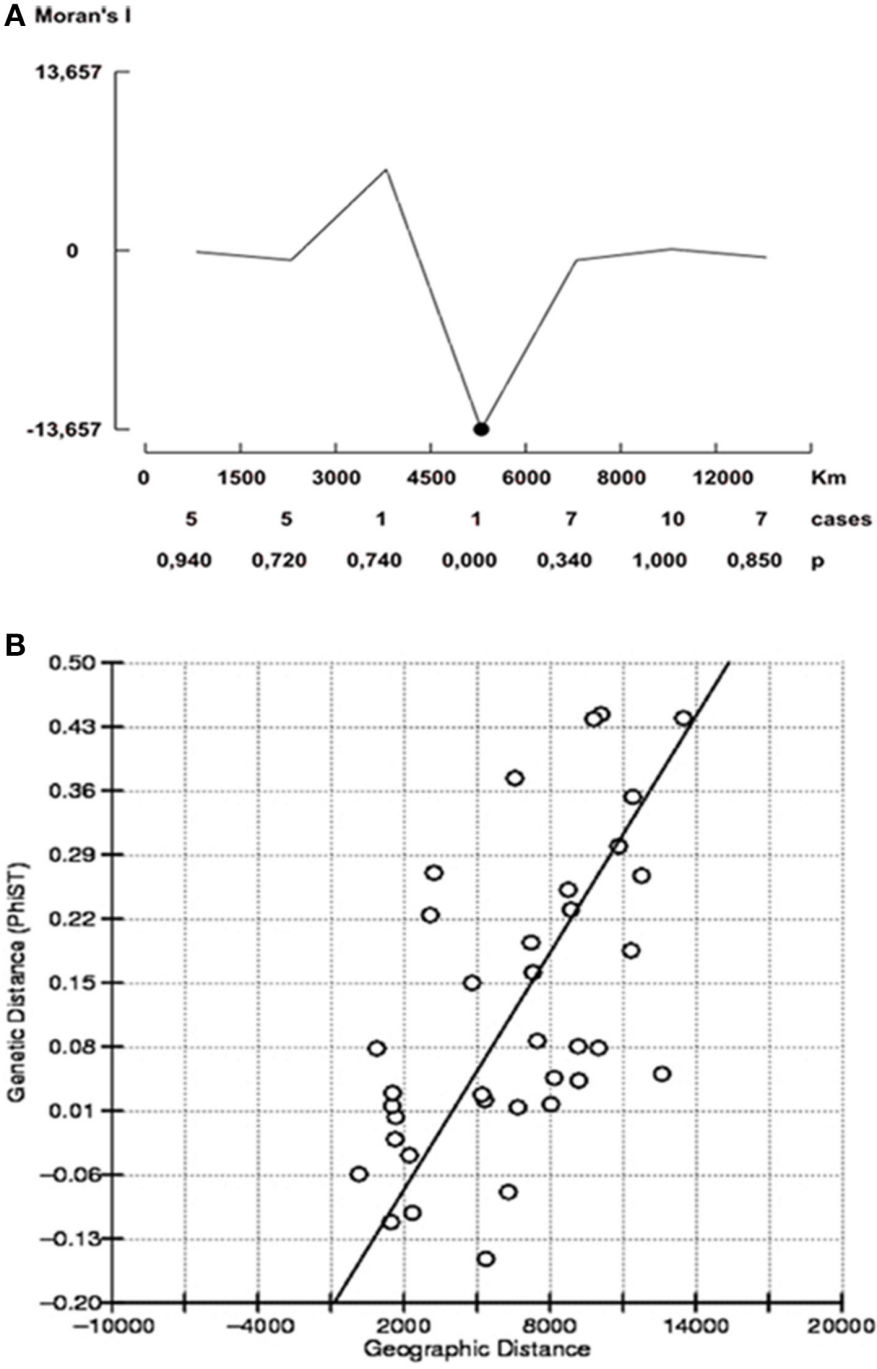
**Spatial analysis. (A)** Moran's correlogram of individual allele frequencies. Moran's *I* was plotted for individual allele frequencies across 6 distance classes (black line). Significant value (black dot) of Moran's *I* indicate positive spatial dependence at *P* < 0.05 **(B)** Mantel test for isolation by distance. Regresion based on genetic distance (PhiF_ST_) values among 9 subpopulations. Regresion slope = 0.0431 ± 0.005; *R*^2^ = 0.350 Mantel probability *P* < 0.01.

Spatial dependence of haplotype frequencies was only detected at the intermediate distance (4500–6000 km) (*P* = 0.000) indicating that the allele frequencies tend to be more different at this geographical distance, and suggesting isolation by distance (Figure 5A). Although not significant Moran's *I* values were observed within the clusters, this value showed a decrease when the pairwise distances increased (data not shown). The partial Mantel test determined that genetic distances and geographical distances among *Y. ruckeri* subpopulations were positively correlated (*Z* = 42.10^*^10^7^; *r* = 0.5915; one-side *P* = 0.0020) (Figure 5B). Similarly, cluster I showed a positive but non-significant correlation (*Z* = 24.23^*^10^8^; *r* = 0.0260; one-side *P* = 0.4690). However, clusters II and III provided evidence of non-significant but negative correlation within the subpopulations (cluster II: *Z* = 14652.4478, *r* = −0.9532; one sided *P* = 1.0000, and cluster III: *Z* = 8189.3193, *r* = −0.3541; one sided *P* = 0.8260) supporting the signal of isolation by distance detected for the overall population.

## Discussion

In this study, biogeographical and inferred dispersal patterns of *Y. ruckeri* population were analyzed on a global scale using multi-sequence data. A pattern of sequence divergence corresponding to geographical area was observed at relatively recent points in the evolutionary history of *Y. ruckeri*. Neighbour network showed a large number of rare haplotypes separated by single-nucleotide differences. Statistical signatures of population expansion were detectable in all subpopulations. Moreover, mismatch distribution indicate that few haplotypes spread and mutated to generate several closely related haplotypes, as is commonly observed during population expansion (Rogers and Harpending, 1992). Furthermore, Tajima's and Fu and Li tests indicated a significant negative deviation from evolutionary neutrality. If *Y. ruckeri* had emerged in one region early in the past and went on to seed the other regions since then (Austin and Austin, 2012), the same haplotypes in all regions would have predominantly collected and signatures of ancient population expansions would not be observed. Conversely, these results revealed that *Y. ruckeri* global population had undergone a population expansion some time in the relative recent past.

This study suggests that the ERM disease emergence in South America and Europe over last few decades resulted from two independent and parallel events. First, an early spread of ERM probably facilitated by human-mediated dispersal, and second, intrinsic factors such as genetic differentiation of *Y. ruckeri* populations, niche specialization and/or isolation by distance.

Although trout farming dates back over 400 years in Europe, about 150 years in the USA, and about 100 in South Africa, *Y. ruckeri* was only isolated for the first time from rainbow trout in the Hagerman Valley of Idaho, USA in 1950s (Busch, 1981). Then, the pathogen was increasingly isolated from other states of the USA and from Canada (Wobeser, 1973). The first report of ERM in Europe was published in 1981 by Lesel et al. (1983), who described the isolation of *Y. ruckeri* from rainbow trout in the southwestern of France. Subsequently, *Y. ruckeri* have been isolated in the 1980s in Denmark, Italy, Norway, UK, and Spain (Austin and Austin, 2012). In South America, reports about the isolation of *Y. ruckeri* from salmonid are limited. The first occurrences of *Y. ruckeri* in Peru were reported from 1998 to 2000 (Bravo and Kojagura, 2004). In Chile, regardless being one of the largest producers of salmonids, the occurrence of ERM was only reported occurring in Atlantic salmon (*Salmo salar*) in 1992 (Toledo et al., 1993). A possible route for the introduction of the pathogen was only described by Michel et al. (1986), who reported in France a clinical case of this bacteriosis in minnows (*Pimephales promelas*) imported from the United States at least since 1981 for live-bait fishing. In addition, as pointed out by Gall and Crandell (1992) and Barnes (2011), different hatcheries may have imported salmonid eggs, fry or brood stocks from infected areas in the absence of strict controls and monitoring schemes. Thus, the pathogen associated with aquaculture animals may have influenced the infectious microbiota and the parasitc fauna of the wild populations (Willumsen, 1989). These works suggested that the worldwide spread of *Y. ruckeri* could have been facilitated by the growth and expansion of aquaculture and other human activities. However, the possibility of *Y. ruckeri* being ubiquitous in the environment, and that aquaculture practices favored its selection and spread cannot be ruled out, despite the fact that the bacterium has never been described in natural fresh water microbial ecology studies.

Analyses of population structure performed in the present study demonstrated a genetic separation among *Y. ruckeri* strains in North America, Europe, and South America. The major ancestral population was formed by isolates from USA, Peru, UK, Finland, and Spain, and isolated mainly from *O. mykiss*. The second ancestral population was defined for the majority of isolates from Chile (isolated from *S. salar*, and belonging to O1b serotype) being more closely related to isolates from UK (from *O. mykiss*, serotype O1b), and in less proportion to some isolates from Portugal (isolated from fish farm sediment and *O. mykiss*, belonging respectively to serotypes O3 and O1a). The third ancestral population was formed by *Y. ruckeri* strains from Canada, Portugal, and Denmark not belonging to the serotype O1a, and with the majority of strains isolated from hosts different to *O. mykiss*. The evidenced genetic admixture in the entire *Y. ruckeri* population also supports the previous hypotheses of specificity of *Y. ruckeri* strains and niche specialization (Bastardo et al., 2012).

The population of *Y. ruckeri* analyzed in this study reflected both signatures of population expansion (a non-equilibrium condition) and isolation by distance (which is consistent with evolutionary equilibrium). This phenomenon may be the result of residual effects of range expansion, and by transfer of strains through countries due to human activities. Humans have had an enormous influence on the global environment and are known to have inflicted genetic variation within other species in the recent past (Conover and Munch, 2002). Recently, the maintenance and spread of *Y. pestis* in Madagascar have been reported as a dynamic and highly active process that relies on the natural cycle between the primary hosts, the black rat, and its flea vectors as well as human activity (Vogler et al., 2011). In *Y. ruckeri* case, it is still likely that sequences reflect mainly a partial return to evolutionary equilibrium after expansion events.

Time from population expansion occurred in a population could be estimated based on the parameter τ (τ = 2μt, where μ is the mutation rate per nucleotide/year) (Slatkin and Hudson, 1991). Without a known mutation rate from *Y. ruckeri* housekeeping genes, it is not possible to accurately pinpoint the time since the inferred expansion events. However, considering that the typical rates of spontaneous mutation per generation in bacteria have been estimated to be on the order of 10^−10^ substitutions per site per year (Drake, 1991), the mutation rate for *Y. ruckeri* (based on 2876 bp) can be preliminary estimated to be on the order of 2.8 × 10^−7^ substitutions per site per year. In theory, if an average of 100–300 generations per year is assumed (Gordon et al., 2002), the time since expansion indicated by mismatch distribution parameter for the overall population of *Y. ruckeri* is at least several thousand years ago (approximately 6375–20,000 years), varying between 800 and 30,000 years among the different areas. This fact suggests ancient spread of *Y. ruckeri* long before to the emergence of modern ERM disease, and highlights the scenario of the independent ERM disease emergence events in the North America and Europe in the last centuries. However, these date ranges should be regarded as preliminary, and further studies are needed on the ancestry and molecular clock in the evolution of *Y. ruckeri* to confirm this hypothesis. Furthermore, the mismatch distributions observed are consistent with expansion in almost all regions, the high values of raggedness index of the Canada, Finland and Portugal subpopulations may indicate a more stable demographic equilibrium with a less sudden expansion (Harpending, 1994).

No evidence for genetic differentiation was obtained in this study to explain the emergence and spread of *Y. ruckeri* biotype 2. However, non-motile isolates were genetically grouped into the same group of motile strains strengthening the theory that biotype 2 has evolved from related motile *Y. ruckeri* strains (Wheeler et al., 2009; Bastardo et al., 2012). The presence of different non-motile haplotypes in the USA, UK, Finland, and Peru support the independent emergence of biotype 2 in geographically separate areas (Ström-Bestor et al., 2010; Bastardo et al., 2012). Furthermore, genetic mixure observed in this study suggests that the emergence of virulent of *Y. ruckeri* variants could be forced by factors extrinsic to the population such as resistance to antibiotics or vaccination.

Selective pressure induced by intensive vaccination could cause changes in phenotypic and immunogenic properties, which can cause outbreaks in vaccinated fish. The observed change from *Y. ruckeri* motile to non-motile isolates being recovered from disease outbreaks could be due to vaccine induced strain replacement (Martcheva et al., 2008). In this context, Pulkkinen et al. (2010) suggested that the presence of several genetically distinct bacterial populations in one area might favor virulence, if the virulent strains have a competitive advantage. The explanation for the increase of biotype 2 *Y. ruckeri* cases in Europe and USA, as well as the emergence of virulent motile isolates belonging to serotypes O1b and O2b in South America could be associated with this evolutionary change.

Few studies have addressed questions of phylogeographic structure and dispersal limitation in bacteria on a truly global scale in discontinuos but globally common habitats (van Gremberghe et al., 2011). Such studies would provide a realistic insight into the degree of dispersal limitation typically encountered by bacteria. In this study, the significant geographic contribution to the overall genetic differences of *Y. ruckeri* was supported by the positive correlation between genetic and geographic distances among strains and populations. The lack of a total geographic structure could be caused by similar nucleotide diversity across spatial scale among the largest subpopulations of USA, Europe and Peru. Nevertheless, geographic isolation of *Y. ruckeri* haplotypes was evidenced in Chile, Canada, and Denmark, which may have epidemiological implications due to differences in clinical outcomes associated with specific genotypes.

In summary, this study provides phylogeographical findings of signature of ancient demographic processes in *Y. ruckeri* population, including spatial expansion, occurring theoretically at least thousands years ago, and more recent genetic divergence among regions. Furthermore, these results suggest that genetic divergence occurring in *Y. ruckeri* populations over last decades are independent, and in some cases isolated events, highlighting the usefulness of genetic studies in explaining the variations in the transmission and maintenance of ERM disease.

## Author contributions

AB, CR, and JR conceived and designed the experiments; AB performed the experiments; AB and JR wrote the paper; CR critically revised the manuscript; AB, CR, and JR edited and approved the manuscript.

### Conflict of interest statement

The authors declare that the research was conducted in the absence of any commercial or financial relationships that could be construed as a potential conflict of interest.
